# Pleuropulmonary Blastoma in a Pediatric Patient: A Case Report and Review of Current Diagnostic and Treatment Strategies

**DOI:** 10.1002/ccr3.70843

**Published:** 2025-09-10

**Authors:** Somayeh Bashiri Aliabadi, Adel Baghersalimi, Bahram Darbandi, Sima Fallah Arzpeyma, Mercedeh Enshaei

**Affiliations:** ^1^ Burn and Regenerative Medicine Research Center Guilan University of Medical Sciences Rasht Iran; ^2^ Pediatric Diseases Research Center Guilan University of Medical Sciences Rasht Iran; ^3^ Department of Radiology, School of Medicine, Poursina Hospital Guilan University of Medical Sciences Rasht Iran

**Keywords:** chemotherapy, pediatric pulmonary tumors, pleuropulmonary blastoma, PPB type III

## Abstract

Pleuropulmonary blastoma (PPB) is a rare, highly aggressive pulmonary tumor that typically presents in the pediatric population. The overall prognosis of PPB is poor, although type I PPB can regress spontaneously. In this case report, the authors present a 3.5‐year‐old boy histopathologically diagnosed with PPB type III, who underwent three courses of the ifosfamide, vincristine, actinomycin D, and doxorubicin (IVADO) chemotherapy regimen after surgical resection. Although the initial post‐chemotherapy evaluations showed no residual or metastatic tumor, based on PET‐CT scan findings, it relapsed 4 months after the last chemotherapy course. Finally, the patient expired due to disease progression. In addition to the case presentation, the authors provide an overview of PPB and discuss how this case illustrates key diagnostic and management challenges in advanced PPB type III.

**Summary**

- Pleuropulmonary blastoma, though rare, should be considered in pediatric patients with persistent respiratory symptoms and abnormal chest imaging.
- Early diagnosis and multidisciplinary treatment significantly improve outcomes.

**Abbreviations**
ASCR: autologous stem cell rescue
CPAM: congenital pulmonary airway malformation
CT: computed tomography
CXR: chest X‐ray
FDG‐PET/CT: fluorodeoxyglucose positron emission tomography‐computed tomography
HDCT: high‐dose consolidation therapy
IHC: immunohistochemistry
IVADO: ifosfamide, vincristine, actinomycin D, and doxorubicin (chemotherapy regimen)
PET‐CT: positron emission tomography‐computed tomography
PICU: pediatric intensive care unit
PPB: pleuropulmonary blastoma

## 1 Introduction

Pleuropulmonary blastoma (PPB) is an infrequent but highly aggressive and malignant tumor that may occur in the lungs and pleura [1]. PPB usually involves children, mainly within the first 4 years of life [2, 3]. It is categorized into three types based on macroscopic findings: type I (cystic), type II (solid and cystic), type III (solid) [3, 4]. It is important to note that not every type I PPB progresses to more malignant forms. In 2006, cystic cases that did not advance were classified as type I regressed (PPB Ir) [5]. Obviously, in the setting of PPB, early histological recognition makes a big difference in the decision‐making for management and prognosis. Our report presents a rare case of type III PPB with post‐chemotherapy recurrence and contributes to the literature by analyzing how this presentation and outcome align with current diagnostic and treatment strategies.

## 2 Case History

A 3.5‐year‐old boy was brought to the pediatric clinic at 17 Shahrivar Hospital in Rasht on June 7, 2022, due to persistent fever, recurrent productive cough, and ongoing breathing difficulties. During the examination, tachypnea was observed, and breath sounds were noticeably absent on the right side. The patient had no complications during the neonatal period, with no history of chronic illness or developmental delay. Antenatal ultrasound examinations were unremarkable, with no evidence of polyhydramnios or congenital pulmonary airway malformation (CPAM). There was also no known family history of pulmonary lesions or childhood tumors.

**FIGURE 1:**
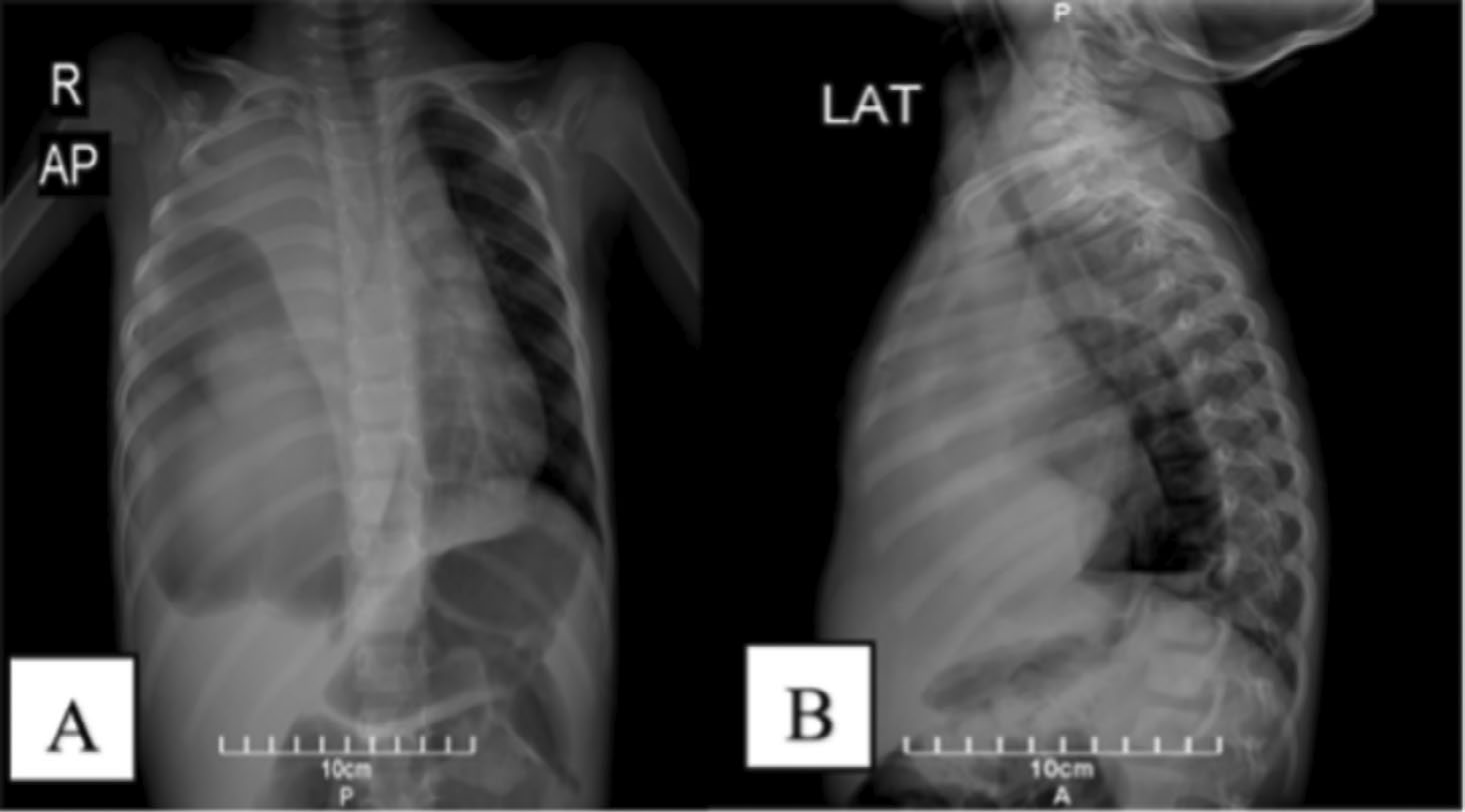
Initial chest radiograph showing two views: (A) Anteroposterior (AP) view, demonstrating diffuse opacification in the right hemithorax, resembling a “white lung,” with blunting of the right costophrenic angle and marked leftward deviation of the heart and mediastinum. A pseudo‐trapping of air is noted in the lower zone of the right lung, with a mass‐like opacification within this area of lucency. (B) Lateral view, further elucidating the mass within the right hemithorax, accompanied by atelectasis in the right upper and lower lobes and a moderate to severe pleural effusion. The combination of these findings results in significant displacement of the mediastinal structures to the left.

The initial chest radiograph demonstrates diffuse opacification of the right hemithorax, with a large air‐fluid level and soft tissue density, causing deviation of the heart and mediastinum to the left side (Figure 1).

### 2.1 Differential Diagnosis, Investigations, and Treatment

**FIGURE 2:**
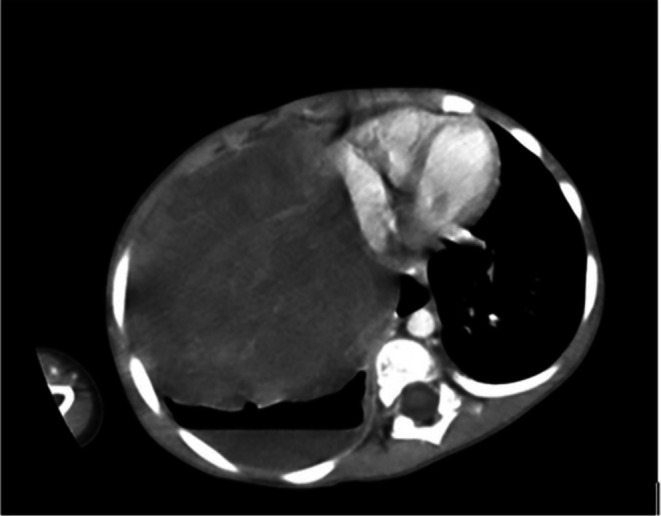
The CT scan reveals a large heterogeneous solid mass measuring 116 × 101 mm with central cystic components in the right hemithorax. An associated air‐fluid level, indicative of hydropneumothorax, is also present. This mass has caused significant atelectasis of the right lung, shifting the heart and mediastinum to the left. Additionally, secondary changes such as increased vascular markings are observed in the left lung. There is no evidence of bony involvement, including the ribs, vertebral bodies, or neural foramina.

The subsequent CT scan demonstrates a large heterogeneous and mixed solid‐cystic mass (116 × 101 mm) in the right hemithorax, involving the upper, middle, and lower lobes. The mass includes a central cystic component, multiple peripheral solid components, and an air‐fluid level (hydropneumothorax). This mass causes severe atelectasis of the right lung, deviation of the heart and mediastinum to the left side, and compressive effects on the right atrium. Prominent vascular markings are observed in the left lung, along with a helium bulge due to vascular engorgement. No signs of rib erosion, chest wall invasion, or intraspinal extension were detected, leading to a probable diagnosis of PPB (Figure 2).

**FIGURE 3:**
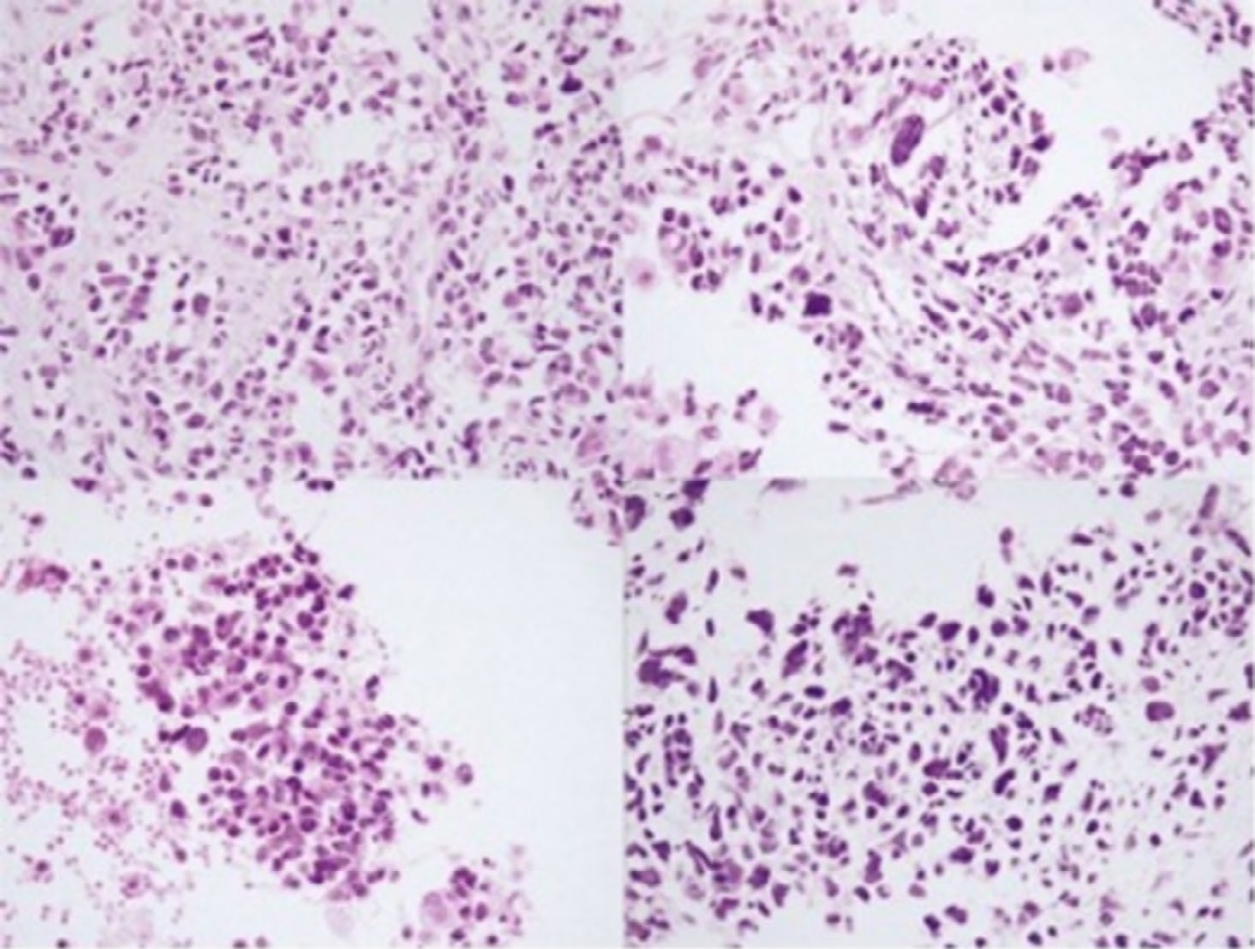
Pathology evaluation revealed neoplastic round, oval, and spindle cells exhibiting significant nuclear pleomorphism, hyperchromasia, and indistinct cell borders. Numerous mitotic figures, including atypical mitoses, were observed. These morphologic features initially suggested a diagnosis of high‐grade sarcoma, with a strong consideration of rhabdomyosarcoma.

At that moment, a CT‐guided core biopsy was performed, and pathology evaluation demonstrated neoplastic round, oval, spindle cells with notable nuclear pleomorphism, hyperchromasia, and the indistinct cell border. Many mitotic figures and some atypical mitoses were observed. Based on morphologic features, high‐grade sarcoma, especially rhabdomyosarcoma, has been suggested initially (Figure 3). However, immunohistochemistry (IHC) was compatible with PPB. Consequently, based on the micromorphological findings, a diagnosis of PPB type III was established. Abdominal ultrasonography was conducted, and commonly co‐occurring cystic nephroma and other tumors associated with PPB were ruled out. As local management, the patient underwent definite surgery. Intraoperative findings demonstrated a huge infiltrated mass in the upper, middle, and lower lobes with an extension to the pleura. Right excision of the lung mass with wide decortication of the right hemithorax was performed. This course of treatment was uneventful. Two weeks after the resection of the mass, adjuvant chemotherapy started with ifosfamide, vincristine, actinomycin D, and doxorubicin (IVADO). The first course was administered on July 5, 2022, the second on July 26, and the third on August 23, each given at approximately 3‐week intervals.

A brain MRI performed on August 18, after the second course, revealed no central nervous system involvement. Following the completion of chemotherapy, an FDG‐PET/CT scan conducted on September 24 showed a complete metabolic response. Metabolically inactive large pulmonary cysts with some solid components and air‐fluid bronchograms in the right hemithorax involving the entire right upper lobe, anterior segment of the right middle lobe, and the posterior segment of the right lower lobes were noted according to the FDG‐PET/CT scan. A follow‐up spiral chest CT scan was conducted on October 13, showing a large (80 × 50 × 45 mm^3^) bulla in the right upper lobe that involved about one‐third of the right hemithorax and caused minimal passive collapse consolidation in adjacent parenchyma. Another finding was a 45 × 27 mm air‐filled lesion with thin septation in the right lower lobe that was noted. A subsequent biopsy of this nodule, performed on December 4, revealed no evidence of metastasis.

### 2.2 Outcome and Follow‐Up

**FIGURE 4:**
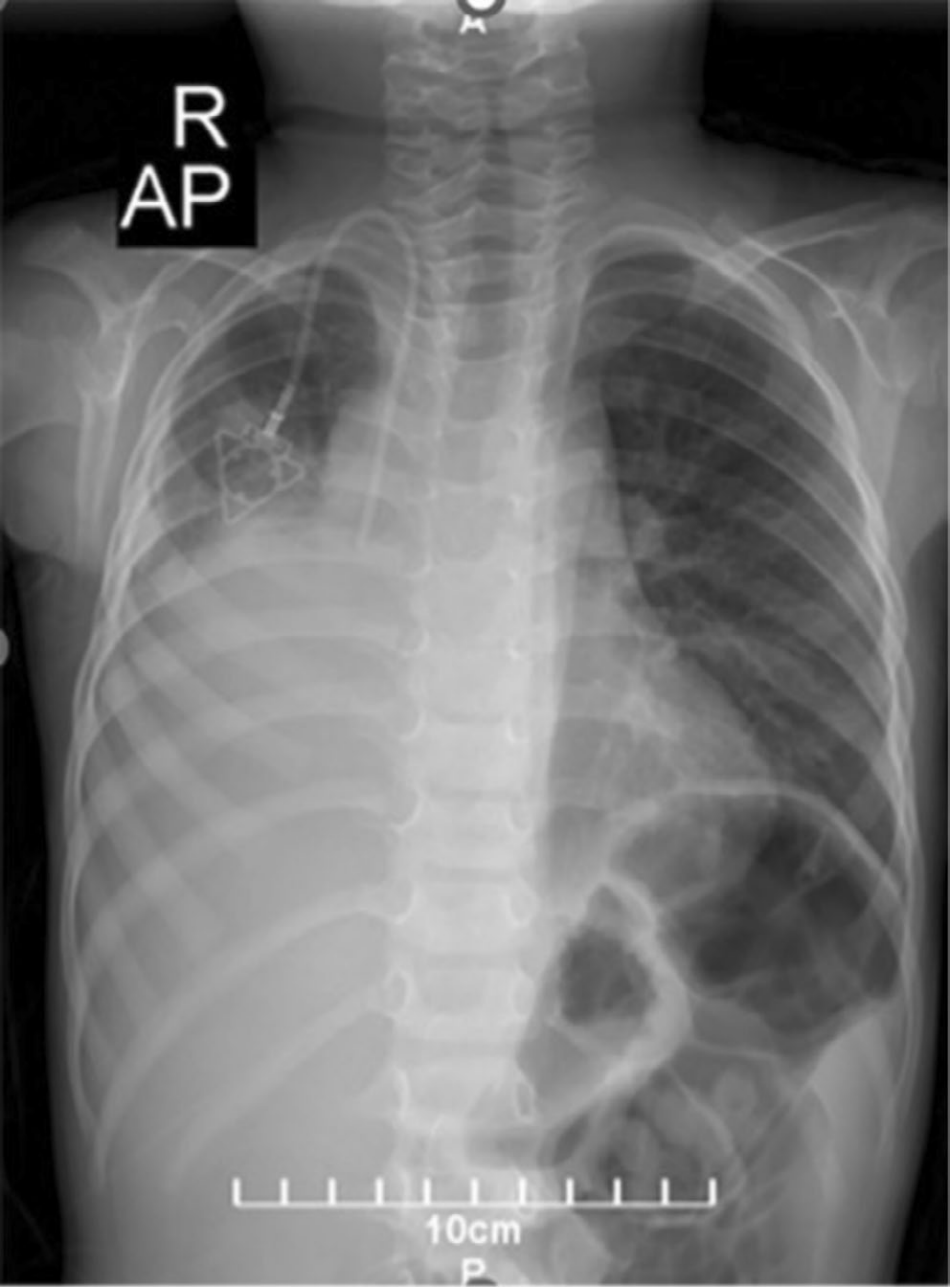
The CXR revealed significant opacification in the right hemithorax, predominantly in the lower zone, without evidence of an air bronchogram or deviation of the trachea or mediastinum.

Nevertheless, 7 months after the final chemotherapy cycle, the cancer relapsed in the previous region. The patient's condition deteriorated, presenting with increased respiratory distress, tachypnea, orthopnea, and a severe cough. A physical examination and CXR (chest X‐ray) revealed pneumothorax in the left hemithorax, necessitating chest tube placement. The CXR revealed significant opacification in the right hemithorax, primarily affecting the lower zone, with no evidence of an air bronchogram or any deviation of the trachea or mediastinum (Figure 4). Subsequently, the patient was admitted to the Pediatric Intensive Care Unit (PICU), where conservative treatment was initiated. On the same admission day, the patient developed bradycardia, leading to a cardiopulmonary arrest. Resuscitation efforts were initiated, but regrettably, the patient did not survive.

## 3 Discussion

### 3.1 Epidemiology and Genetic Basis

PPB is a potentially life‐threatening malignant tumor that almost always occurs in children and comprises 15% of all primary pediatric pulmonary neoplasms, with 25% in a constitutional or familial setting. PPB originates from the pleuropulmonary and extrapulmonary intrathoracic mesenchyme [3, 6, 7, 8]. According to the Manivel et al. study and World Health Organization in 2015, in contrast with the biphasic epithelial‐stromal morphology of the classic adult type, PPB in children is categorized as mesenchymal neoplasms [9, 10].

It is estimated that two‐thirds of patients diagnosed with PPB neoplasm will be identified to have a DICER1 gene mutation in germline cells. The DICER1 gene mutation increases the risk for other hereditary neoplasms, including cystic nephroma, pituitary blastoma, embryonal rhabdomyosarcomas, ovarian sex cord‐stromal tumors, and other tumors. Therefore, the DICER1 gene should be screened in a patient diagnosed with PPB for related tumors [4, 11, 12, 13, 14, 15].

In our case, DICER1 mutation testing was not performed due to the patient's rapid clinical decline and the limited availability of timely genetic testing resources at our center.

PPB mostly appears in the first 4 years of life in children. Nevertheless, rare occurrences have been declared beyond the first decade of life, and few cases of PPB have been represented in adolescents and young adults. Purely cystic PPB type may be diagnosed in the first year of life without any symptoms or in the setting of a spontaneous pneumothorax [3, 16].

### 3.2 Clinical Presentation and Diagnosis

PPB may be presented with chest pain, fever, difficulty in breathing, cough, hemoptysis, weakness, and neurological symptoms due to brain metastasis. However, patients show different symptoms according to the subtypes: In type I, air‐filled cysts compressing on airways lead to respiratory distress with or without pneumothorax. Patients suffering from type II and III tumors may present with pneumonia or other symptoms such as dyspnea and chest pain, cough, fever, difficulty in breathing, malaise, weakness, and anorexia. A chest wall deformity or a tension pneumothorax may be seen in asymptomatic lesions [17, 18, 19].

### 3.3 Classification and Prognosis

PPB is exclusively characterized by distinct morphologic types: Type I (cystic), Type Ir (type I regressed), Type II (combined solid and cystic), and Type III (solid). PPB type I may progress to the aggressive solid type III within the first 4–5 years of life. Moreover, high‐grade sarcoma components with a mixed pattern may be seen in the solid component of both type II and III [4, 16, 20].

Type I PPB has the most favorable prognosis among all subtypes of PPB with a 91% 5‐year overall survival rate. Although Type I PPB may progress to the aggressive Type II and Type III PPB, it may also regress to Type Ir. “r” stands for regressing without any malignant cells [5, 21, 22].

Type Ir is a purely cystic tumor added as a subtype of PPB in 2006, which occurs mainly at 48 months old. The histopathological characteristic of this subtype is cystic areas with few spindle cells and foci of dystrophic calcification that lack a primitive cell component [5].

Type II PPB has both cystic and solid components. The median age at diagnosis for type II is 35 months, and the 5‐year survival rate is 71%. On histopathology, solid nodules and polypoid tissue may be present within the cystic growth. Type III PPB is a solid tumor with the poorest prognosis among all types of PPB (53% survival rate within 5 years). The median age for type III PPB is 44 months. In histopathological evaluation, it has a completely solid component. A combination of blastic and sarcomatous components compose solid tissue in type II and III PPB [3, 5].

### 3.4 Imaging and Diagnostic Approach

The first imaging modality applicable for PPB evaluation is chest radiography. The PPB neoplasm usually appears on the right side of the lung for unknown reasons, as seen in our patient. Due to the nonspecific manifestation of respiratory distress, the lesion is usually neglected in the early stage, and due to a delay in diagnosis, it can cause complete infiltration of the involved hemithorax with contralateral tracheal and mediastinal deviation [14]. This can lead to an incorrect diagnosis of massive pleural effusion based on chest radiograph findings [5]. Other patterns that can be commonly seen are partial opacification of the lung, benign‐appearing cystic lung disease, pleural effusion, tension pneumothorax, and mediastinal shift [23]. The tumor may occur in different sizes located peripherally in the lung with or without the involvement of the pleura or chest wall. A complex cyst, such as a cyst with a solid component or a mass partially filling the thorax, indicates type II or type III PPB, respectively [2, 18, 24], whereas type I lesions appear as a single cyst or multicystic lesions with fluid or air within [14, 23].

Contrast‐enhanced chest CT aids in evaluating the tumor's morphology, enhancement pattern, and extent. Aforementioned data help in differentiating PPB from other possible differential diagnoses such as neuroblastoma and CPAM. In our case, heterogeneous right hemithorax and mixed solid cystic mass indicated a type III PPB confirmed by CT‐guided core biopsy. Moreover, contrast‐enhanced CT studies of both the chest and abdomen are necessary for staging patients suspected of PPB. Patients with type II and type III PPB require additional brain MRI and bone scan for staging, since in these types, metastasis mostly involves the central nervous system and bones [5, 14, 23]. In our study, brain MRI and FDG‐PET/CT scan were conducted, and no distant metastasis was detected.

### 3.5 Treatment Strategies

Surgical excision and adjuvant chemotherapy are the treatment for type I PPB, but type Ir tumors can be followed without chemotherapy. In contrast to types I and Ir, which have a relatively good prognosis, type II and type III tumors require aggressive surgery and chemotherapy. Moreover, neoadjuvant chemotherapy should be considered in cases with large tumors to reduce the tumor size, followed by surgical resection. Finally, additional radiation therapy is recommended in patients with nonresectable tumors, residual tumors, and cases with brain metastases. High‐dose consolidation therapy (HDCT) with autologous stem cell rescue (ASCR) is also recommended for recurrent tumors [5, 23].

### 3.6 Case Comparison With Literature

Our patient's clinical course reflects several patterns reported in the literature. The diagnosis of Type III PPB at 3.5 years aligns with the median age of 44 months reported by the International PPB Registry [4]. The tumor's right‐sided location is consistent with known right‐sided predominance [14]. Initial complete metabolic response after IVADO chemotherapy was achieved, yet the disease relapsed approximately 7 months later. This mirrors the recurrence risk documented by Schultz et al., who reported only a 53% 5‐year survival for Type III cases, despite multimodal treatment [23]. These findings underscore the aggressive nature and relapse potential of this subtype.

## 4 Conclusion

PPB is a rare and aggressive tumor primarily affecting children, with a dismal prognosis, particularly in cases of type III PPB. This case report emphasizes the challenges in managing PPB and the crucial significance of early diagnosis. Despite advancements in the diagnosis and treatment of PPB, the prognosis remains challenging, especially in advanced cases. Ongoing research aims to improve therapies and comprehend the genetic factors linked with PPB, offering hope for enhanced survival rates. Vigilance among medical professionals is imperative in the approach to PPB, emphasizing the necessity of early detection and personalized treatment strategies in combating this rare and aggressive tumor.

## Author Contributions

**Somayeh Bashiri Aliabadi:** writing – original draft, writing – review and editing. **Adel Baghersalimi:** conceptualization, writing – review and editing. **Bahram Darbandi:** writing – review and editing. **Sima Fallah Arzpeyma:** writing – review and editing. **Mercedeh Enshaei:** writing – review and editing.

## Ethics Statement

The study protocol was approved by the institutional ethics committee.

## Consent

Written informed consent was obtained from the patient's parents for publication of this case report and accompanying images.

## Conflicts of Interest

The authors declare no conflicts of interest.

## Data Availability

The data that support the findings of this case report are available from the corresponding author upon reasonable request. All data have been anonymized to protect patient confidentiality.

## References

[1] R. Calabria , M. S. Srikanth , K. Chamberlin , J. Bloch , and J. B. Atkinson , “Management of Pulmonary Blastoma in Children,” American Surgeon 59, no. 3 (1993): 192–196. 8476159

[2] A. A. Khan , A. K. El‐Borai , and M. Alnoaiji , “Pleuropulmonary Blastoma: A Case Report and Review of the Literature,” Case Reports in Pathology 2014 (2014): 509086. 25177506 10.1155/2014/509086 PMC4142298

[3] J. T. Stocker , A. N. Husain , and L. P. Dehner , “Pediatric Tumors,” in Dail and Hammar's Pulmonary Pathology Vol. II, 3rd ed. (Springer, 2008).

[4] Y. H. Messinger , D. R. Stewart , J. R. Priest , et al., “Pleuropulmonary Blastoma: A Report on 350 Central Pathology‐Confirmed Pleuropulmonary Blastoma Cases by the International Pleuropulmonary Blastoma Registry,” Cancer 121, no. 2 (2015): 276–285. 25209242 10.1002/cncr.29032 PMC4293209

[5] P. K. Madaan , H. S. Sidhu , S. Girdhar , and K. K. Mann , “Pleuropulmonary Blastoma: A Report of Three Cases and Review of Literature,” Radiology Case Reports 16, no. 10 (2021): 2862–2868. 34401014 10.1016/j.radcr.2021.06.046 PMC8349909

[6] S. G. Hamad , A. Al‐Naimi , and M. Abu‐Hasan , “Pleuropulmonary Blastoma (PPB) in Child With DICER1 Mutation: The First Case Report in the State of Qatar,” Case Reports in Pulmonology 2021 (2021): 1983864. 34745680 10.1155/2021/1983864 PMC8570905

[7] G. Bisogno , S. Sarnacki , T. Stachowicz‐Stencel , et al., “Pleuropulmonary Blastoma in Children and Adolescents: The EXPeRT/PARTNER Diagnostic and Therapeutic Recommendations,” Pediatric Blood & Cancer 68, no. S4 (2021): e29045. 33826235 10.1002/pbc.29045 PMC9813943

[8] J. L. Huret , M. Ahmad , M. Arsaban , et al., “Atlas of Genetics and Cytogenetics in Oncology and Haematology in 2013,” Nucleic Acids Research 41 (2013): D920–D924. 23161685 10.1093/nar/gks1082 PMC3531131

[9] J. C. Manivel , J. R. Priest , J. Watterson , et al., “Pleuropulmonary Blastoma. The So‐Called Pulmonary Blastoma of Childhood,” Cancer 62, no. 8 (1988): 1516–1526. 3048630 10.1002/1097-0142(19881015)62:8<1516::aid-cncr2820620812>3.0.co;2-3

[10] M. Mlika , F. Anjum , and F. El Mezni , Pleuropulmonary Blastoma (StatPearls Publishing, 2023). 30480950

[11] A. J. Bauer , D. R. Stewart , J. Kamihara , et al., “DICER1 and Associated Conditions: Identification of At‐Risk Individuals and Recommended Surveillance Strategies‐Response,” Clinical Cancer Research 25, no. 5 (2019): 1689–1690. 30824630 10.1158/1078-0432.CCR-18-3495

[12] L. S. Hiemcke‐Jiwa , S. van Belle , A. Eijkelenboom , et al., “Pleuropulmonary Blastoma (PPB) and Other DICER1‐Associated High‐Grade Malignancies Are Morphologically, Genetically and Epigenetically Related—A Comparative Study of 4 PPBs and 6 Sarcomas,” Annals of Diagnostic Pathology 60 (2022): 152002. 35779311 10.1016/j.anndiagpath.2022.152002

[13] D. G. Sabapathy , R. P. Guillerman , R. C. Orth , et al., “Radiographic Screening of Infants and Young Children With Genetic Predisposition for Rare Malignancies: DICER1 Mutations and Pleuropulmonary Blastoma,” American Journal of Roentgenology 204, no. 4 (2015): W475–W482. 25794098 10.2214/AJR.14.12802

[14] J. P. Lichtenberger, 3rd , D. M. Biko , B. W. Carter , M. A. Pavio , A. R. Huppmann , and E. M. Chung , “Primary Lung Tumors in Children: Radiologic‐Pathologic Correlation From the Radiologic Pathology Archives,” Radiographics 38, no. 7 (2018): 2151–2172. 30422774 10.1148/rg.2018180192

[15] J. R. Priest , J. Watterson , L. Strong , et al., “Pleuropulmonary Blastoma: A Marker for Familial Disease,” Journal of Pediatrics 128, no. 2 (1996): 220–224. 8636815 10.1016/s0022-3476(96)70393-1

[16] I. A. González , D. R. Stewart , K. A. P. Schultz , A. P. Field , D. A. Hill , and L. P. Dehner , “DICER1 Tumor Predisposition Syndrome: An Evolving Story Initiated With the Pleuropulmonary Blastoma,” Modern Pathology 35, no. 1 (2022): 4–22. 34599283 10.1038/s41379-021-00905-8 PMC8695383

[17] J. C. Salazar , M. Pardo , and V. M. Mora‐Bautista , “Pleuropulmonary Blastoma: Case Report,” Archivos Argentinos de Pediatría 116, no. 3 (2018): e455–e458. 29756723 10.5546/aap.2018.e455

[18] J. R. Priest , M. B. McDermott , S. Bhatia , J. Watterson , J. C. Manivel , and L. P. Dehner , “Pleuropulmonary Blastoma: A Clinicopathologic Study of 50 Cases,” Cancer 80, no. 1 (1997): 147–161. 9210721

[19] A. Addanki , K. Chaitanya , S. Bartakke , and S. Sethuratnam , “A Case Report of Pleuropulmonary Blastoma Presenting as Tension Pneumothorax,” Indian Journal of Medical and Paediatric Oncology 38, no. 1 (2017): 70–72. 28469342 10.4103/0971-5851.203515 PMC5398112

[20] J. R. Priest , D. A. Hill , G. M. Williams , et al., “Type I Pleuropulmonary Blastoma: A Report From the International Pleuropulmonary Blastoma Registry,” Journal of Clinical Oncology 24, no. 27 (2006): 4492–4498. 16983119 10.1200/JCO.2005.05.3595

[21] K. Landry‐Truchon , N. Houde , M. Lhuillier , et al., “Deletion of Yy1 in Mouse Lung Epithelium Unveils Molecular Mechanisms Governing Pleuropulmonary Blastoma Pathogenesis,” Disease Models & Mechanisms 13, no. 12 (2020): dmm045989. 33158935 10.1242/dmm.045989 PMC7790197

[22] L. P. Dehner , Y. H. Messinger , K. A. Schultz , G. M. Williams , K. Wikenheiser‐Brokamp , and D. A. Hill , “Pleuropulmonary Blastoma: Evolution of an Entity as an Entry Into a Familial Tumor Predisposition Syndrome,” Pediatric and Developmental Pathology 18, no. 6 (2015): 504–511. 26698637 10.2350/15-10-1732-OA.1 PMC9743680

[23] K. A. P. Schultz , A. K. Harris , A. T. Nelson , et al., “Outcomes for Children With Type II and Type III Pleuropulmonary Blastoma Following Chemotherapy: A Report From the International PPB/DICER1 Registry,” Journal of Clinical Oncology 41, no. 4 (2023): 778–789. 36137255 10.1200/JCO.21.02925 PMC9901992

[24] N. G. Ordóñez , “The Immunohistochemical Diagnosis of Mesothelioma: A Comparative Study of Epithelioid Mesothelioma and Lung Adenocarcinoma,” American Journal of Surgical Pathology 27, no. 8 (2003): 1031–1051. 12883236 10.1097/00000478-200308000-00001

